# Usefulness of Respiratory Stability Time-Guided Management to Prevent Readmission of Chronic Heart Failure Patients at Home: A Multicenter, Single-Arm, Open-Label Clinical Study (ITMETHOD-HF-III)

**DOI:** 10.3390/jcm14134653

**Published:** 2025-07-01

**Authors:** Teruhiko Imamura, Yasuhiro Akazawa, Shunsuke Saito, Yasushi Sakata, Shigeru Miyagawa, Tomomi Yamada, Hidetsugu Asanoi, Koichiro Kinugawa

**Affiliations:** 1 Second Department of Internal Medicine, University of Toyama, Toyama 930-0194, Japan; 2 Department of Cardiovascular Medicine, Graduate School of Medicine, Osaka University, Suita 565-0871, Japan; 3 Department of Cardiovascular Surgery, Graduate School of Medicine, Osaka University, Suita 565-0871, Japan; 4 Department of Medical Innovation, Osaka University Hospital, Suita 565-0871, Japan; 5 Toyama Nishi General Hospital, Toyama 939-2716, Japan

**Keywords:** telemonitoring, congestion, heart failure, hemodynamics

## Abstract

**Background**: Telemonitoring aimed at detecting subclinical heart failure and facilitating medication up-titration offers a promising approach to reducing heart failure hospitalizations. Our team has recently developed a non-invasive metric called “respiratory stability time (RST)”, which quantifies respiratory instability, a surrogate marker of subclinical worsening heart failure. A decrease in RST below 20 s predicts the onset of worsening heart failure within 28 days. However, the clinical utility of RST-guided management in reducing mortality and heart failure hospitalizations remains uncertain. **Methods**: The Innovative Tele-Monitoring Environment To Halt Ongoing Deterioration of Heart Failure-III (ITMETHOD-HF-III) is a non-blinded, interventional, multicenter, single-arm study. Eighty heart failure patients with a history of at least two prior hospitalizations for heart failure will be enrolled. After validating the robustness of RST measurements, participants will be monitored for 1.5 years through daily RST measurements. Mandatory up-titration of heart failure medications will be started if RST values decrease below 20 s for two consecutive days or decrease progressively below 30 s over 10–90 days from RST values above 45 s maintained for over 1 month, irrespective of the presence of heart failure signs/symptoms. Medication adjustment will continue until RST exceeds 30 s. The study will compare a composite endpoint of heart failure hospitalization and cardiac death between the present RST-guided group and a historical control group from the ITMETHOD-HF-II trial, in which management was based on patients’ symptoms. **Results:** We anticipate that the precent ITMETHOD-HF-III study will demonstrate that mandatory, RST-guided heart failure management significantly reduces the incidence of the primary composite endpoint—heart failure hospitalization and cardiac death—compared with symptom-guided standard care in the historical control group (ITMETHOD-HF-II). **Conclusions**: The ITMETHOD-HF-III study aims to demonstrate the clinical efficacy of RST-guided management in reducing heart failure hospitalization rates and cardiac mortality by enabling early detection of subclinical heart failure and facilitating timely medication adjustments, irrespective of heart failure signs/symptoms. If successful, RST-guided management could establish a new standard for telemonitoring heart failure patients in outpatient settings.

## 1. Introduction

Despite the widespread adoption of established pharmacological treatments for heart failure, the morbidity and mortality rates of heart failure patients remain unacceptably high [1]. Notably, readmission due to worsening heart failure is a strong predictor of subsequent hospitalizations and increased mortality [2]. Recurrent hospitalizations are not only associated with adverse clinical outcomes, but they also contribute to a diminished quality of life and increased healthcare costs. Thus, preventing heart failure-related hospitalizations is a critical strategy in mitigating the global burden of this condition [3].

A major challenge in reducing hospitalizations lies in the early identification of worsening heart failure to enable timely optimization of medical therapy [4]. Typically, patients seek medical attention at outpatient clinics when they experience symptoms of heart failure, such as dyspnea on exertion [5]. Clinicians then evaluate heart failure severity using physical examinations, chest radiography, and plasma B-type natriuretic peptide measurements. However, the heart failure often progresses insidiously, preceding the onset of overt symptoms [6]. Consequently, by the time symptoms become evident, the condition has frequently advanced to a stage where hospitalization is unavoidable [7].

Telemonitoring has attracted significant attention as a promising tool for tracking heart failure progression and detecting early signs of heart failure deterioration before symptom onset [8]. Conventional parameters such as blood pressure, heart rate, respiratory rate, and body weight are easily measurable without specialized equipment but lack disease specificity, limiting their utility in early detection of heart failure exacerbations [9].

Several advanced modalities have been proposed to enhance telemonitoring capabilities. Thoracic impedance monitoring, for example, has been used in patients with cardiac resynchronization therapy devices [10]. However, its application is restricted to individuals with implantable cardiac devices, and its high false-positive rate necessitates concurrent evaluation of additional variables for accurate assessment [11].

Another notable innovation is the CardioMEMS system, which involves implanting a sensor in the pulmonary artery to continuously monitor pulmonary artery pressure [12]. In the CHAMPION trial, CardioMEMS-guided therapy significantly reduced heart failure hospitalizations by enabling earlier intervention based on pulmonary artery pressure trajectory, rather than relying solely on clinical symptoms or weight changes [13]. However, the invasive nature of sensor implantation and the associated high costs pose significant barriers to widespread adoption, particularly in regions where the device is not yet available, such as Japan [14].

**Figure 1:**
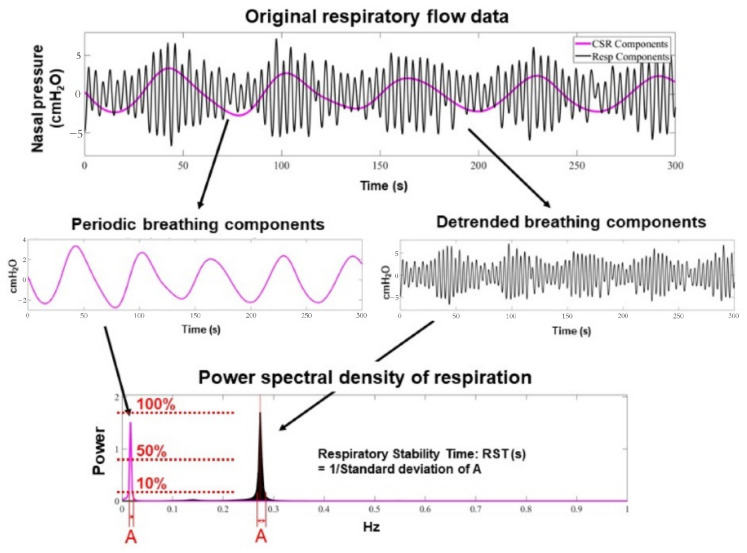
Methodology to calculate RST values (reused with permission) [18]. All spectral power is normalized by the power spectral density or the ratio of the maximum power of the components. All respiration frequency points with a power spectral density > 10% are equally adopted in the assessment of respiratory instability. Very low-frequency points of the periodic breathing curve are only adopted if the power spectral density of the very low-frequency component is >50% of the maximum power of the respiratory component. Respiratory frequency points are evaluated using standard deviation, and RST value is defined as the inverse of the standard deviation. CSR, Cheyne–Stokes respiration; Resp, respiration; RST, respiratory stability time.

Recently, our team introduced a novel, non-invasive metric termed “**Respiratory Stability Time (RST)**”, which quantifies respiratory instability (Figure 1) [15]. Respiratory instability, such as Cheyne–Stokes respiration or irregular rapid shallow breathing without periodicity, reflects underlying neuro-hormonal, hemodynamic, and respiratory derangements associated with worsening heart failure. Using a proprietary algorithm, we developed an automated system to calculate RST.

Previous studies have demonstrated that patients with RST values below 20 s exhibit significantly worse 1-year clinical outcomes than those with higher RST values [15]. The IMIZUNO-HOME trial, which incorporated telemonitoring of multiple parameters, including RST, showed that RST independently predicted the onset of heart failure exacerbations [16]. During index hospitalizations due to heart failure, RST improvements correlated with the resolution of heart failure-related congestion [17]. In another study, therapeutic interventions such as transcatheter aortic valve replacement for severe aortic stenosis were shown to ameliorate RST levels [18].

Building on these findings, a multicenter, prospective study, **Innovative Tele-Monitoring Environment To Halt Ongoing Deterioration of Heart Failure-I (ITMETHOD-HF-I)**, was conducted from 2017 to 2019 [19]. This study used an upgraded RST monitoring system and identified an RST threshold of 20 s as the optimal cut-off for predicting future heart failure-related hospitalizations.

Subsequently, the **ITMETHOD-HF-II trial**, a multicenter, prospective, randomized, controlled study, evaluated the clinical utility of prospective RST monitoring. Patients were advised to visit outpatient clinics if their RST fell below 20 s, although the decision to adjust medications was left to the discretion of the attending physicians. Clinical decisions eventually relied on traditional signs and symptoms assessed during outpatient visits despite continuous RST monitoring.

To fully elucidate the clinical implications of RST-guided management, particularly its potential to detect “occult” heart failure progression before symptom onset, give us a chance to enhance heart failure medications, and prevent worsening heart failure, therapeutic intervention must be systematically aligned with RST alerts “alone”, irrespective of heart failure signs/symptoms. The current **ITMETHOD-HF-III study** addresses this gap by “mandating” the up-titration of heart failure medications whenever RST reaches the alert threshold, irrespective of clinical signs or symptoms. This single-arm, multicenter, prospective study will compare outcomes, including heart failure hospitalizations and cardiac mortality, against those observed in ITMETHOD-HF II, where medication adjustments were discretionary.

## 2. Materials and Methods

### 2.1. Patient Selection

**Figure 2:**
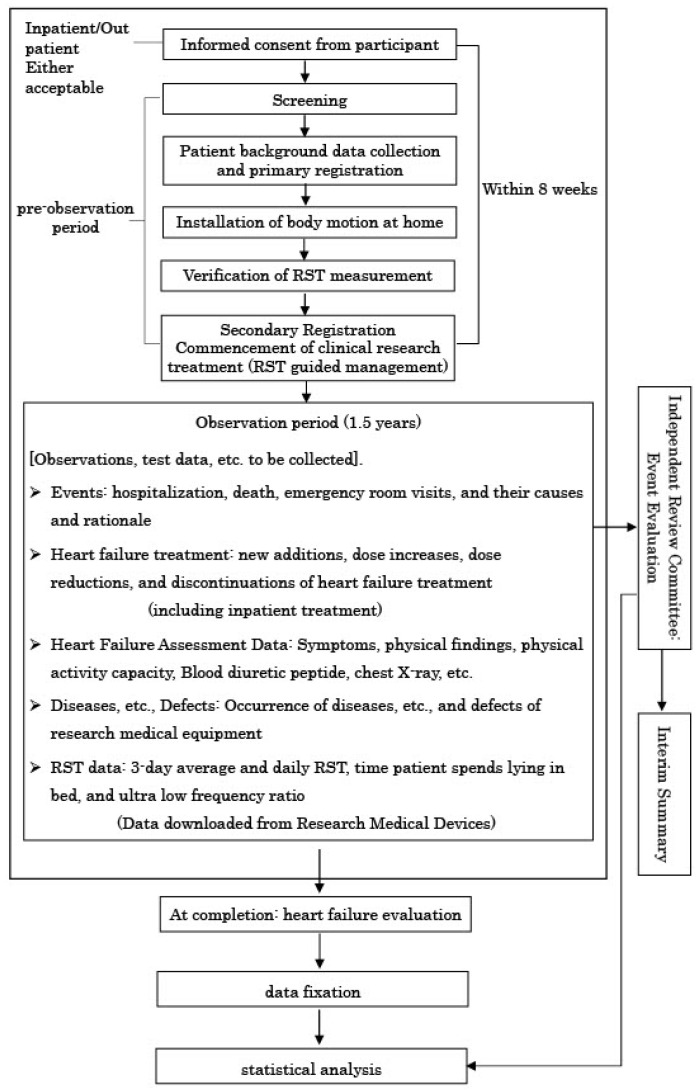
Registration and follow-up route. Following obtaining written, informed consent and screening, patients are registered in this study (primary registration). Following validation of RST measurement, patients are formally registered, and RST monitoring is started (secondary registration). RST, respiratory stability time.

Patients who fulfill all inclusion criteria and meet none of the exclusion criteria will be eligible for enrollment in this study (Table 1). Participants from the previous ITMETHOD-HF-II trial will also be included as a historical control group for comparative analysis. The patient selection process will follow a two-step registration system, as illustrated in Figure 2.

(1) Primary registration

Patients with chronic heart failure and a history of at least two prior hospitalizations for heart failure will qualify for primary registration. However, individuals with conditions that may affect RST measurements, including a history of stroke, the use of mechanical circulatory support devices, or a diagnosis of obstructive sleep apnea syndrome, will be excluded at this stage.

(2) Secondary registration

Patients listed in the primary registration will undergo RST measurement trials. Only those whose RST can be measured accurately will advance to secondary registration. Upon inclusion in the secondary registration, these patients will begin RST-guided telemonitoring.

### 2.2. Study Design

After obtaining informed consent, patients will undergo a screening process to determine their eligibility (Figure 2). Eligible patients will be enrolled in the primary registration phase, during which the telemonitoring system will be set up, and initial RST measurements will be conducted. Upon verification of accurate RST measurements, patients will progress to secondary registration. At this stage, RST-guided management will start.

Clinical parameters, including RST values, will be continuously monitored over a 1.5-year observation period. The historical control group will consist of participants from the prior **ITMETHOD-HF II trial**, in which RST values were monitored, but therapeutic decisions were left to the discretion of attending physicians. In contrast, the current study “mandates” therapeutic interventions whenever RST values fall below the alert threshold, irrespective of clinical signs or symptoms.

### 2.3. RST Measurement

**Figure 3:**
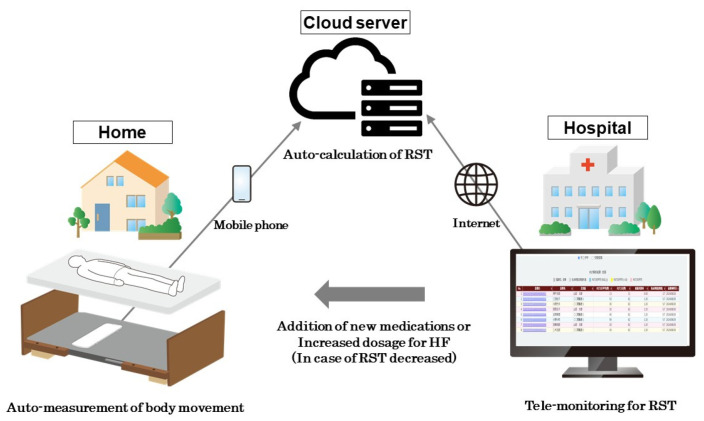
Entire RST telemonitoring system between home, cloud server, and hospital. All respiratory signals are obtained and transferred to a cloud server. The all-night RST value is automatically calculated on the server until the next morning. The researchers in each institute monitor RST values every day remotely by reviewing data on the cloud server. HF, heart failure; RST, respiratory stability time.

The telemonitoring system used in this study comprises a piezoelectric, non-contact sensor (Nemuri SCAN, Paramount Bed Co., Ltd., Tokyo, Japan) positioned beneath the bed sheet, connected to a microcomputer that serves as an Internet-enabled gateway installed in the patient’s home (Figure 3) [19]. This system continuously collects respiratory signals and monitors the patient’s time spent lying in bed.

During the study period, respiratory signals are captured nightly at a sampling frequency of 16 Hz, spanning the entire duration of the patient’s sleep. These signals are transmitted to a cloud server, where all-night RST values are calculated and stored (Figure 3) [15]. To standardize the analysis, data collected during the fixed hours of 23:00 to 5:00 are used. All respiratory signals transmitted to the cloud are automatically processed and analyzed using an RST calculation program (HeartLab, Inc., Kobe, Japan) by 8:30 am every morning.

**Figure 4:**
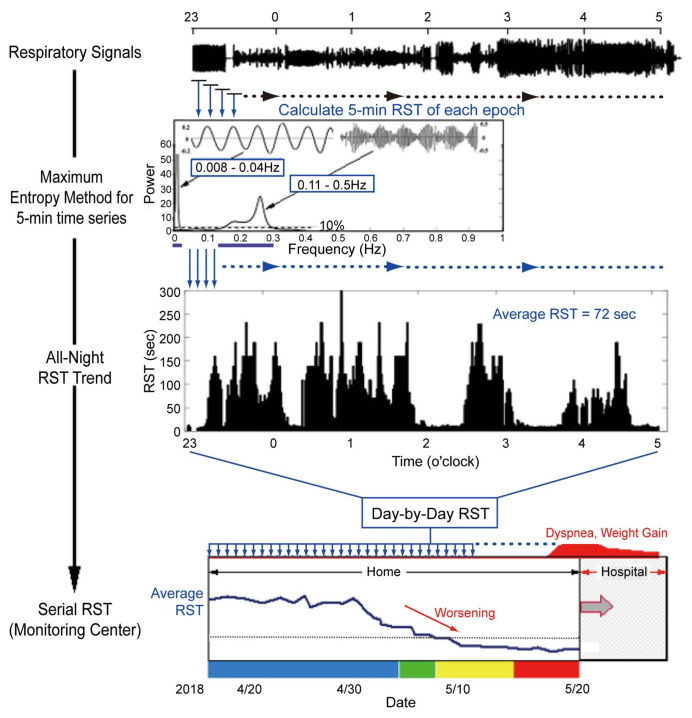
Calculation of all-night RST values in a cloud server (reused with permission) [19]. Breathing signals to calculate all-night RST are collected from 23:00 to 5:00 o’clock of the next morning every day. In the pre-processing steps for RST measurement, we focus on two frequency ranges: high-frequency respiratory components (0.11–0.5 Hz) and very low-frequency components corresponding to the periodic breathing (0.008–0.04 Hz). These two signals are divided into serial 5 min segments every 50 s. For each epoch of the 5 min high- and very low-frequency data, power spectra are serially calculated with the maximum entropy method and normalized as a percentage of the maximum power of the respiratory components. The high-frequency distribution for spectral power > 10% of the maximum and the very low-frequency distribution for spectral power > 50% of the maximum are adopted to calculate a standard deviation of respiratory frequency variations. The RST value is defined as the reciprocal of the standard deviation. For each epoch, RST value is serially calculated and averaged to represent all-night respiratory instability. RSTs are graded as colors acconrding their severity: blue, normal; green, mild; yellow, moderate; red, severe. RST < 20 s is assigned to yellow and is a threshold of alert. RST, respiratory stability time.

The detailed methodology for RST measurement has been previously described [15]. As part of the pre-processing pipeline (Figure 4), the direct current component of the signals is removed to eliminate zero-frequency impulses, and a zero-phase digital filter is applied to isolate respiratory signals by excluding high-frequency components above 0.5 Hz. Subsequently, the processed signals are resampled at 4 Hz. To estimate RST, two frequency ranges are analyzed as previously established.

(1) **Respiratory frequency components**

These are derived from instantaneous respiratory signals after the exclusion of high- and low-frequency noise using a 5th-order bandpass Butterworth filter with cut-off frequencies of 0.11 Hz and 0.5 Hz.

(2) **Very low-frequency components**

These correspond to periodic breathing patterns and are obtained by tracing the peaks of instantaneous respiratory signals, adjusting the baseline to zero, and applying a bandpass filter with cut-off frequencies of 0.008 Hz and 0.04 Hz.

For serial analysis of all-night RST, respiratory signals are divided into consecutive 5 min segments, updated every 50 s. A minimum of 420 segments (≥350 min) of data are required for analysis each night. For each segment, the **maximum entropy method** is applied to extract specific respiratory and periodic breathing components from the spectral data.

Spectral power is normalized as a percentage of the maximum respiratory power. Respiratory frequency points with spectral power exceeding 10% of the maximum respiratory power are considered in the evaluation of respiratory instability. For periodic breathing components, only very low-frequency points with maximum power exceeding 50% of the respiratory components’ maximum power are included in the RST calculation.

For each epoch, the standard deviation of the respiratory frequency distribution is calculated, and RST is defined as the reciprocal of this standard deviation. All-night RST values are then averaged to provide a single representative measure of nightly respiratory instability.

**Figure 5:**
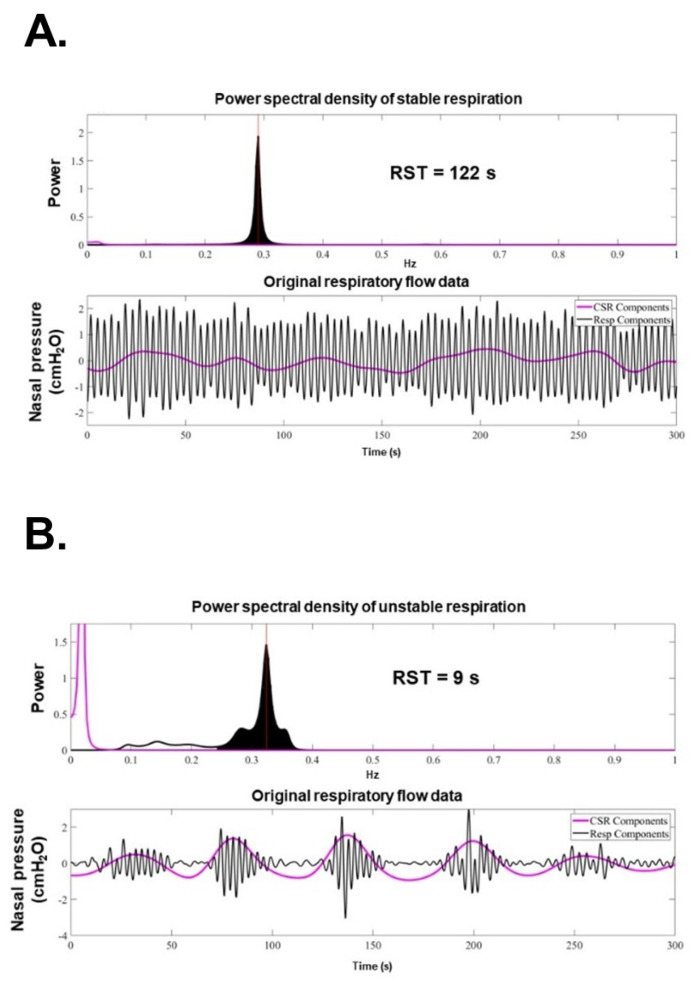
Representation of high RST (**A**) and low RST levels (**B**) (reused with permission) [18]. In stable periodic breathing, the frequency spectrum is narrowly distributed, and the RST value is high (**A**). In unstable periodic breathing, the spectral components are widely distributed and include very low-frequency components, resulting in a low RST value. Cheyne–Stokes respiration; Resp, respiration RST, respiratory stability time.

RST values are updated daily, and a 3-day moving average is calculated to monitor trends. These trends are visualized on the monitoring center’s dashboard, where representative high and low RST values are displayed (Figure 5A,B).

### 2.4. RST-Guided Management

**Figure 6:**
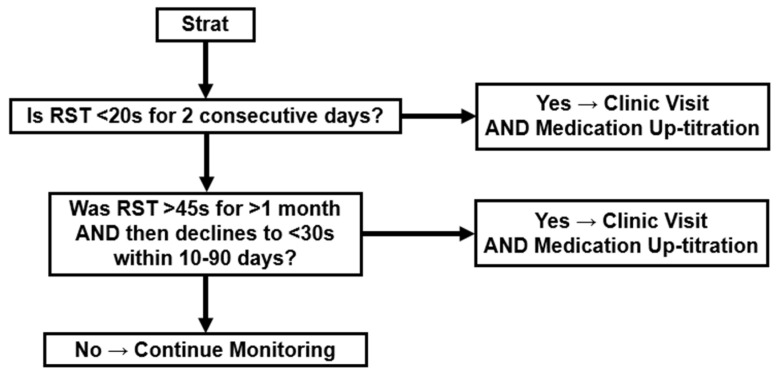
A flowchart illustrating the logic behind RST alert thresholds. Patients are advised to visit the outpatient clinic and undergo medication up-titration if (1) RST falls below 20 s for two consecutive nights, or (2) RST previously maintained above 45 s for over one month subsequently decreases below 30 s within a 10–90 day period. If neither condition is met, patients continue regular telemonitoring.

Therapeutic intervention is initiated by up-titrating heart failure medications when daily RST meets either of the following alert thresholds for two consecutive days, irrespective of the presence of heart failure symptoms: (1) the RST value falls below 20 s for two consecutive days; or (2) the average RST value remains above 45 s for over one month but then decreases progressively to below 30 s within a period of 10 to 90 days. A visual decision tree outlining the RST alert criteria and subsequent actions is summarized in Figure 6.

Patients are instructed to visit the outpatient clinic within three business days when their RST reaches the alert threshold. If, despite therapeutic intervention, the RST does not improve to exceed 30 s, patients will be asked to revisit the outpatient clinic for further evaluation and intensification of treatment. This process will be repeated as necessary until the RST stabilizes above 30 s. Management will continue to ensure sustained improvement in RST levels.

### 2.5. Study Visits and Follow-Up

Following informed consent and initial screening, patients are enrolled in the primary registration. During screening, data are collected on heart failure symptoms, vital signs, chest X-ray, electrocardiogram, laboratory parameters (plasma B-type natriuretic peptide or serum NT-pro B-type natriuretic peptide, hemoglobin, serum creatinine, and blood urea nitrogen), transthoracic echocardiography, and medication history (Table 2). On confirmation of appropriate RST measurement, patients are included in the secondary registration, and RST-guided management is started.

Throughout the 1.5-year observation period, patients are monitored at the outpatient clinic at regular intervals, typically once per month. Clinical data collected at each visit are recorded when available. In the event of hospitalization due to low RST, chest X-ray and laboratory tests are mandatory, and the dosage of heart failure medications is systematically up-titrated, regardless of the presence of heart failure symptoms.

### 2.6. Primary and Secondary Outcomes

The primary outcome is defined as a composite of heart failure hospitalization and cardiac death. Heart failure hospitalization is characterized by an admission due to worsening heart failure requiring significant up-titration of diuretic dosage, intravenous diuretic administration, or mechanical circulatory support, with a minimum of 24 h of in-hospital observation.

The secondary outcomes include feasibility, efficacy, and exploratory outcomes. The feasibility of RST telemonitoring is assessed. Efficacy outcomes encompass recurrent heart failure hospitalization, cardiac death, the occurrence of heart failure hospitalization or cardiac death within 60 days following RST decrease, heart failure hospitalization or cardiac death in patients with RST improvement (versus no improvement), heart failure hospitalization or cardiac death in patients with RST decrease (versus no decrease), and the increase of RST within one month following RST decrease. As an exploratory outcome, heart failure hospitalization or cardiac death in patients without RST decrease is also evaluated. All events will be evaluated by the independent committee.

### 2.7. Sample Size Calculations

From the previously conducted ITMETHOD-HF-II trial, 52 of the 73 patients who met the eligibility criteria for the present study will be included as the historical control group. The heart failure admission rate during the 1.5-year observation period was 43.1%. The rate of preventing heart failure admissions through RST guidance is estimated to be 50%, leading to an estimated heart failure admission rate of 21.6% in the RST-guided group.

The Lakatos method indicated that a sample size of 73 patients is required to demonstrate significance with a two-tailed *p*-value of 5% and a power of 25% using the log-rank test. To account for potential dropouts and withdrawals, the required sample size was determined to be 80 patients. As a sensitivity analysis, we also calculated the required sample size assuming a smaller, more conservative 30% relative risk reduction (from 43.1% to 30.2%). Under the same statistical conditions, the Lakatos method estimates that 146 patients would be required. While the current study remains powered to detect a 50% reduction, this additional analysis highlights the impact of varying effect sizes on required sample size and will inform future confirmatory trial design.

### 2.8. Statistical Analysis

All statistical analyses will be performed using SAS Version 9.4 (SAS Institute Inc., Cary, NC, USA), with a two-sided significance level set at *p* < 0.05 to indicate significance. Continuous variables will be presented as medians with interquartile ranges, and categorical variables will be expressed as frequencies and percentages. The Mann–Whitney U test will be used to compare continuous variables between the two groups, and the chi-squared test will be applied for categorical variables.

For the primary outcome, event rates will be estimated using the Kaplan–Meier method, and their 95% confidence intervals will be calculated using Greenwood’s formula. The log-rank test will be used for comparisons between groups. A Cox proportional hazards model will be used to estimate the hazard ratio of the RST-guided group compared with the historical control group. Potential confounding variables will be statistically adjusted for if significant differences in baseline characteristics are observed between the two groups to minimize selection bias. For secondary outcomes, the Andersen–Gill model will be applied to recurrent event data, and a mixed-effects logistic model will be applied to events occurring within 60 days after RST decline. Other secondary endpoints will be analyzed using the same methods as the primary endpoint.

### 2.9. Ethical Considerations

This is a non-blinded, interventional, multicenter, single-arm trial designed to evaluate the clinical benefits of RST-guided management in patients with chronic heart failure. The study has been registered with jRCT (registration number: jRCTs042240196) and will be conducted in accordance with the Declaration of Helsinki and the International Conference on Harmonization Good Clinical Practice guidelines. The ethical aspects of the study plan, protocol, and informed consent process have been approved by the Clinical Research Review Board, University of Toyama (SCR2024002) on 29 January 2025 prior to the commencement of the study. All participants will provide written informed consent before being enrolled in the study.

## 3. Expected Results

We anticipate that the ITMETHOD-HF-III study will demonstrate that mandatory, RST-guided heart failure management significantly reduces the incidence of the primary composite endpoint—heart failure hospitalization and cardiac death—compared with symptom-guided standard care in the historical control group (ITMETHOD-HF-II). Specifically, we expect that the implementation of early therapeutic interventions triggered solely by RST alerts, irrespective of clinical signs or symptoms, will enable the timely optimization of pharmacologic therapy and effectively prevent clinical decompensation.

It is projected that patients receiving RST-guided management will show a lower cumulative incidence of first heart failure hospitalization and cardiac death over the 1.5-year follow-up period. Moreover, secondary analyses are expected to reveal that the magnitude and trajectory of RST recovery following intervention will correlate with improved clinical outcomes, while persistent low RST values despite treatment may identify patients at elevated risk for adverse events. In particular, we expect that patients who achieve an RST increase to ≥30 s within 14 days after the alert threshold will exhibit favorable prognoses.

Feasibility assessments are expected to confirm the reliability and clinical usability of the automated, contactless RST monitoring system in real-world outpatient settings. We also anticipate that the study will validate the prognostic utility of RST as a non-invasive surrogate for pulmonary congestion and subclinical hemodynamic deterioration.

If the results support our hypothesis, this study will provide the first evidence that mandatory therapeutic intervention based solely on RST alerts can reduce heart failure events, thereby establishing a novel, scalable paradigm for telemonitoring-guided care in chronic heart failure management. Whether such an improvement of clinical outcomes by the RST-guided management may improve the cost-effectiveness of heart failure management remains a future concern.

## 4. Discussion

The innovative technology developed by our team facilitates the comprehensive measurement of overnight physiological variables in a fully automated manner, eliminating the need for attaching or implanting biological sensors onto patients. A sheet-type sensor continuously monitors and records respiratory patterns throughout the night. The acquired data undergoes complete automated processing and is transmitted to a cloud server via the Internet, where all-night RST is computed each morning.

In this ITMETHOD-HF-III study, the aim is to evaluate the clinical significance of RST-guided management in reducing hospitalization rates and mortality associated with heart failure, compared with conventional symptom-guided management strategies. Notably, this approach “mandates” the up-titration of heart failure medications in patients whose RST falls below a predefined threshold, regardless of the presence or absence of overt heart failure symptoms (probably absence in many cases).

### 4.1. How to Detect Sub-Clinical Worsening Heart Failure

Worsening heart failure typically begins with an increase in intracardiac filling pressures [20]. Such increases are often detectable several weeks before the emergence of overt signs and symptoms of heart failure. To monitor these initial and sometimes trivial changes, direct daily measurement of pulmonary artery pressure using wireless implantable monitoring devices has been used [12]. For instance, the CHAMPION trial demonstrated that heart failure management guided by wireless pulmonary artery hemodynamic monitoring effectively reduced heart failure hospitalizations through timely adjustments of medications [13]. However, these devices are hindered by significant drawbacks, including their invasiveness and substantial medical costs.

Our technology for calculating RST values addresses these limitations through its non-invasive nature, affordability, and broad applicability, maintaining predictive accuracy [15]. Within the human lung, the progression of pulmonary congestion is inherently monitored by four built-in sensor systems, particularly the vagal nerve collagen sensors. The irritant vagal afferents activated by lung stretch reflexes respond to pulmonary congestion by exerting counteracting effects on respiratory patterns, leading to respiratory instability. In addition, increased central blood volume mechanically restricts lung inflation, resulting in rapid and shallow breathing. Consequently, elevated cardiac filling pressures and central blood volume can be reliably inferred from decreases in RST values, well before the clinical manifestation of heart failure.

Supporting this, prior studies have shown that an RST decrease below 20 s was a robust predictor of future heart failure hospitalization, with detection occurring up to three weeks prior to clinical onset [19].

### 4.2. Rationale for the Inclusion/Exclusion Criteria

Optimal patient selection is essential for the effectiveness of RST-guided management. The primary goal of this intervention is to prevent hospitalizations due to heart failure. Patients with a heightened risk of hospitalization are ideal candidates for RST-guided management, whereas those with minimal risk may not derive significant benefit from such monitoring [21]. Therefore, the inclusion criteria require participants to have a documented history of at least two prior hospitalizations for heart failure.

Conversely, patients with advanced or refractory disease may not be suitable for RST-guided management, since their conditions are less likely to respond to any therapeutic adjustments [22]. To address this, we exclude individuals receiving durable left ventricular assist devices, as well as those with persistently low RST values below 20 s, which signify severe and unresponsive respiratory instability.

In addition, patients with conditions that could confound RST measurements or limit their applicability are excluded. These conditions include sleep apnea syndrome, advanced pulmonary diseases, and prior stroke [19]. Though these exclusion criteria are necessary to maintain the integrity of the study, they also highlight the current limitations of RST in certain patient populations.

### 4.3. How to Demonstrate the Clinical Implication of RST-Guided Management

Evidence from previous studies suggests that a decrease in RST below 20 s can predict heart failure hospitalization up to 28 days in advance [19]. However, the clinical efficacy of aggressive therapeutic interventions to prevent such hospitalizations in real-world daily practice remains uncertain.

In the ITMETHOD-HF-II trial, patients were encouraged to be admitted when their RST fell below 20 s. However, therapeutic decisions, including the up-titration of heart failure medications, were left to the discretion of attending physicians. In many cases, medications were not adjusted, likely because patients were asymptomatic due to early hospital admission. To unequivocally demonstrate the utility of RST in identifying subclinical heart failure and preventing hospitalizations, “mandatory” up-titration of heart failure medications is essential when RST values fall below this threshold.

Findings from the ITMETHOD-HF-II study reinforce this approach. All patients who achieved RST values of ≥30 s within 14 days by therapeutic intervention successfully avoided heart failure hospitalizations. This is a rationale why we set 30 s of RST as a therapeutic target. Based on these findings, we strongly propose repeating therapeutic adjustments until RST values exceed 30 s.

However, mandatory interventions carry potential risks, particularly in patients with hypovolemia or renal impairment, in whom hemodynamic instability may occur. Therefore, careful risk-benefit analyses are crucial to ensure the safety of mandatory interventions, especially in asymptomatic patients.

It is also important to recognize that not all participants may experience an increase in RST despite aggressive therapeutic interventions. A thorough assessment of patient characteristics, the types of interventions attempted, and clinical outcomes is necessary to identify refractory cohorts. These findings will help inform the development of tailored therapeutic strategies for these populations in future studies.

### 4.4. Study Limitation

This study protocol has several limitations that warrant consideration. First, the ITMETHOD-HF-III study is designed as a single-arm, non-randomized trial utilizing a historical control group from the previous ITMETHOD-HF-II study. Although this approach facilitates rapid implementation and comparative analysis, it inherently limits the ability to control for unmeasured confounders and time-dependent biases such as changes in clinical practice, therapy use, and healthcare delivery. Differences in clinical practices, patient management strategies, or healthcare delivery systems between the two study periods may influence outcomes independently of the intervention itself. We may add propensity score analysis to further address residual confounding.

Second, the protocol mandates therapeutic up-titration solely based on RST values, irrespective of heart failure symptoms or physical findings. While this strategy aims to validate the utility of RST in detecting subclinical deterioration, it also introduces a potential risk of overtreatment, particularly in patients with borderline RST values or comorbidities such as renal dysfunction or hypovolemia. Final decision to strengthen the therapy, including the up-titration of diuretic dose, is at the discretion of the attending physicians. The response of RST values to each therapeutic intervention also remains a concern to be analyzed. The safety and tolerability of such aggressive, symptom-independent interventions remain to be fully established.

Third, RST measurement relies on a piezoelectric sensor and proprietary algorithm, which, although validated in previous studies, may be influenced by factors such as sleep position, body movement, and comorbid pulmonary or neurological conditions. We defined several stringent inclusion and exclusion criteria to mitigate these confounders. Conversely, such strict criteria may limit generalizability to real-world clinical management. Further studies are warranted to evaluate the applicability of this system and our therapeutic strategy utilizing this technology.

Fourth, due to the open-label, single-arm design, neither participants nor clinicians are blinded to the intervention, which may introduce bias in outcome ascertainment and therapeutic decision-making. Although outcome adjudication is performed in a blinded manner by an independent committee, the potential for performance and detection bias cannot be fully excluded.

Fifth, as the present manuscript is a study protocol, baseline demographic, clinical, and instrumental characteristics—such as comorbid conditions, risk factor profiles, and heart failure phenotypes—are not yet available and thus not presented. These parameters are known to affect prognosis, therapeutic responses, and risk of heart failure readmission. We will thoroughly incorporate these data and adjust them, if applicable, in subsequent publications upon study completion.

Sixth, the sample size calculation was based on an anticipated 50% reduction in the event rate, which may be relatively optimistic. Although this assumption was informed by the early predictive performance of RST observed in prior studies—comparable to or exceeding that of invasive monitoring technologies such as CardioMEMS—we acknowledge that the actual effect size may be smaller. The results of this study will provide critical data for refining effect size estimates and conducting sensitivity analyses in future confirmatory trials.

Lastly, although the study design includes frequent follow-up and protocolized management pathways, patient adherence to outpatient visits following RST alerts is critical. Delays or non-compliance in clinic attendance may reduce the effectiveness of the intervention and confound outcome assessment.

Despite these limitations, the ITMETHOD-HF-III study represents an important step toward validating a novel, non-invasive, and scalable tool for the early detection and management of worsening heart failure in the outpatient setting.

## 5. Conclusions

This study highlights the potential of RST-guided management as an innovative, non-invasive approach to predict and prevent heart failure hospitalizations. By identifying “subclinical” heart failure through automated RST monitoring, early therapeutic interventions can be implemented to mitigate disease progression. The study also emphasizes the necessity of “mandatory” therapeutic interventions when RST values decline below a predefined threshold to fully realize the clinical utility of RST-guided management. Future efforts should focus on refining therapeutic strategies, assessing refractory cohorts, and optimizing the integration of RST-guided management into broader heart failure care protocols following the detailed assessment of our findings.

## Figures and Tables

**Table 1:** Inclusion and exclusion criteria.

**Eligibility Criteria**	
	Primary registration
	(1) Patients who understood the study and gave informed consent
	(2) Patients aged ≥20 years
	(3) Patients who satisfied the guideline’s definition of heart failure
	(4) Patients with at least two heart failure hospitalizations within the past five years
	(5) Patients who can be followed at outpatient clinic
	(6) Patients who had heart failure symptoms with NYHA functional class II–III
	Secondary registration
	Of patients who were included in the primary registration, patients whose RST could be measured at home
**Exclusion Criteria**	
	Primary registration
	(1) Patients who sleep together with others
	(2) Patients who do not sleep between 23:00 and 5:00
	(3) Patients with significant obstructive pulmonary disease
	(4) Patients requiring a respiratory support device during sleep
	(5) Pregnant patients
	(6) Patients with significant obstructive sleep apnea syndrome
	(7) Patients with histories of stroke
	(8) Patients who receive or will receive a ventricular assist device
	(9) Patients dependent on hemodialysis
	(10) Patients with significant renal or liver impairment
	(11) Patients participating in other interventional trials
	(12) Patients with cognitive impairment
	(13) Patients who cannot be followed during the observation period
	(14) Other patients who are inappropriate for the study
	Secondary registration
	(1) Patients with inappropriate RST measurement both during the first and second weeks
	a. <5 days per week of RST measurements
	b. Mean RST during a week <20 s or 60 s
	(2) Patients who are hospitalized following primary registration
	(3) Patients who are inappropriate for the study

NYHA, New York Heart Association; RST, respiratory stability time.

**Table 2:** Timing of clinical data collection.

Observation/Inspection			Informed Consent	Previous Observation			Secondary Registration	Observation Period (78 Weeks)					
				Screening	Primary Registration	RST Measurement		Medical Examination When RST Decreases	Revisit After Decreased RST Visit	Periodic Medical Check-Up	Non-Scheduled Visit	During Exacerbation of Heart Failure	At the Time of Termination (At the Time of Cancellation) *^1^
Allowance		Days since second registration	−56 to −8		−22 to −8	−21 to 0	0	―	―	―	―	―	516 to 576
		Number of days since RST decline	―			―	―	0 to 7	−21	―	―	―	―
Informed consent			●										
Participant registration					●		●						
Participant background				●									
Heart Failure Assessment	Symptoms			●				●	●	Δ	Δ	●	●
	Physical examination findings			●				●	●	Δ	Δ	●	●
	Chest X-ray			●				● *^2^	Δ *^2^	Δ *^2^	Δ *^2^	● *^2^	●
	ECG			●				Δ	Δ	Δ	Δ	Δ	●
	Blood test	BNP (NT-ProBNP)		●				● *^2^	Δ *^2^	Δ *^2^	Δ *^2^	● *^2^	●
		Hb		●				●	Δ	Δ	Δ	●	●
		Cre, BUN		●				●	Δ	Δ	Δ	●	●
	Echocardiography *^3^			●				Δ	Δ	Δ	Δ	Δ	Δ
	Determination of the need for inpatient care							●	●	●	●	●	●
Heart failure treatment		Baseline		●									
		Change						●	Δ	Δ	Δ	●	Δ
Body motion sensor: provided (installed)					●								
Body motion sensor: take back													●
RST measurement					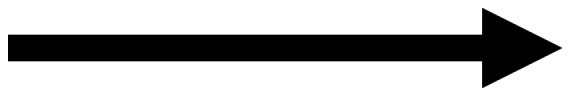			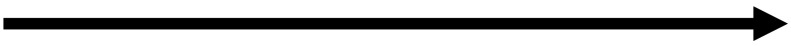					
Clinical research treatment implementation								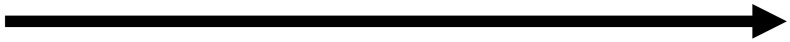					
Confirmation of disease, etc., and failure					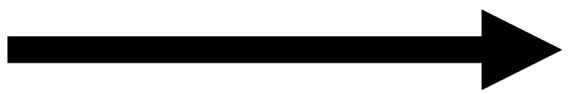			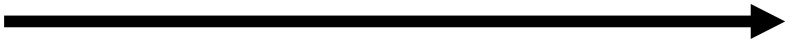					

●: implementation, Δ: data collection if implemented. *^1^ At the time of discontinuation, perform heart failure evaluation prior to discontinuation whenever possible. *^2^ Either chest X-ray and BNP (NT-ProBNP) can be performed; both BNP and NT-ProBNP are acceptable, but they should be standardized during the clinical study period in the same study participants. *^3^ For echocardiography, data within 1 year prior to obtaining consent are acceptable. Black arrows represent continuation of interventions. BNP, B-type natriuretic peptide; BUN, blood urea nitrogen; Cre, creatinine; ECG, electrocardiogram; Hb, hemoglobin; NT-ProBNP, N-terminal pro B-type natriuretic peptide; RST, respiratory stability time.
